# Does a Good Firm Breed Good Organizational Citizens? The Moderating Role of Perspective Taking

**DOI:** 10.3390/ijerph16010161

**Published:** 2019-01-08

**Authors:** Byung-Jik Kim, Mohammad Nurunnabi, Tae-Hyun Kim, Se-Youn Jung

**Affiliations:** 1 Sogang Business School, Sogang University, Seoul 04107, Korea; kimbj82@business.kaist.edu; 2 St Antony’s College, University of Oxford, 62 Woodstock Road, Oxford OX2 6JF, UK; 3 Department of Accounting, Prince Sultan University, P.O. Box 66833, Riyadh 11586, Saudi Arabia; 4 College of Business, Korea Advanced Institute of Science and Technology, Seoul 02455, Korea; taehyun@kaist.ac.kr; 5 Prime College, Korea National Open University, Seoul 03087, Korea

**Keywords:** corporate social responsibility, organizational citizenship behavior, organizational commitment, employees’ perspective taking, moderated mediation model

## Abstract

Although some previous studies have examined the impact of corporate social responsibility (CSR) on employees in an organization, they have mainly focused on employees’ perceptions or attitudes rather than behaviors. However, in that employees’ behaviors are the direct outcome of the perceptions or attitudes and critically affect organizational outcomes, we need to investigate the impact of CSR on employees’ behaviors. Based on the context-attitude-behavior framework, we investigate the underlying process of the association between CSR and employees’ behavior with a moderated mediation model. Specifically, we hypothesize (1) the intermediating effect of organizational commitment (OC) in the association between CSR and organizational citizenship behavior (OCB) and (2) the contingent role of employees’ perspective taking ability (PT) in the CSR-OC link. Using three-wave survey data from 301 currently working employees in Korea, we found that OC mediates the association between CSR and OCB and that PT can positively moderate the CSR-OC link. Our findings suggest that OC (as an intermediating process) and PT (as a contingent factor) function as important underlying mechanisms to elaborately describe the CSR-OCB link.

## 1. Introduction

As a firm’s morality has become a central issue in business activities, it is natural for firms to emphasize the importance of corporate social responsibility (CSR) [1,2,3,4]. Although some studies have reported that the relationship between CSR and organizational outcomes is not significant and even negative [5,6,7,8], the majority of studies have demonstrated that CSR functions as an effective strategy to increase competitive advantage of companies [9,10], improving organizational outcomes such as financial performances of firms [11,12].

Even though many studies have investigated the CSR-organizational outcomes link, we believe there have been research gaps which remain unsolved. First, while many existing studies have examined the effect of CSR on macro-level organizational outcomes (e.g., financial performances, product quality, reputation of firm, and consumer loyalty), they tend to underexplore the relationship between CSR and organizational outcomes at the individual level (e.g., employee’s perceptions, attitudes, and behaviors) [2,13,14]. However, given that employees are the actors who not only actually implement organizational strategy, but also translate moral practices into organizational outcomes [13,14], employees’ perceptions, attitudes, and behaviors in an organization are critical in explaining organizational outcomes. Thus, investigating the individual-level outcomes of CSR would be meaningful.

Second and more importantly, while some studies have examined the relationship between CSR and organizational outcomes at the individual level, they have mainly focused on perceptions or attitudes of employees including organizational commitment, organizational identification, and job satisfaction [15,16,17,18,19,20], relatively ignoring employees’ behaviors. Perceptions or attitudes are very important, but they would be ultimately expressed in the form of their behaviors. In other words, behaviors are more directly and closely associated macro-level outcomes such as financial performances rather than the perceptions or attitudes. Thus, we argue that it is meaningful to examine the impact of CSR practices on employees’ behaviors.

Lastly, several scholars argue that more work needs to explore underlying mechanisms (i.e., mediators and moderators) of the association between CSR and organizational outcomes at the individual level [2,13,14]. The existing studies have mainly examined the bilinear relationship between CSR and outcomes such as organizational commitment, organizational identification, job satisfaction, and in-role/extra-role performance [2,13,14]. In this study, by establishing a moderated mediation model, we attempt to extend our understanding of the underlying mechanisms that drive the effect of CSR on organizational outcome to the individual level.

To complement the above research gaps, in the present research, we focus on organizational citizenship behavior (OCB) as an important employee behavior. OCB is defined as “individual behavior that is discretionary, not directly or explicitly recognized by the formal reward system, and that in the aggregate promotes the effective functioning of the organization” [21] (p. 4). The concept has been known to be closely related to both individual-level outcomes and organizational-level outcomes [22,23,24].

To elaborately explain underlying mechanisms of the CSR-OCB link by relying on a theoretical ground, we first consider organizational commitment (OC) as a mediating factor which connect the CSR with OCB. The mediating mechanism is based on a context–attitude-behavior framework [25,26], which suggests that organizational contexts such as various kinds of systems, rules, and practices in an organization, substantially create employees’ attitudes and behaviors. In specific, CSR practices may function as an important social context, and thus, it may affect employees’ attitudes (i.e., OC) and subsequently their behaviors (i.e., OCB) [25,26,27]. For example, existing studies suggest that CSR positively affects organizational commitment of individual employees by increasing their perceived obligation to repay, based on social exchange process [15,18,19,20]. Also, many empirical findings support that the more employees commit to their organizations, the more they show organizational citizenship behaviors because employees with high level of OC tend to make extra efforts to achieve the collective goal of their organization [28,29,30,31,32]. Therefore, we argue that CSR would increase OCB by enhancing employee’s OC.

Also, we investigate the effect of an employee’s perspective taking ability as a moderator on the CSR-OC link. Perspective taking is defined as a cognitive procedure in which a person tries to understand others’ thoughts, intentions, preferences, and values from the viewpoint of those [33]. Employees’ perspective taking is the beginning of the interpretation but also critically affects the processes of the interpretation [33,34]. Thus, it is likely to affect sense-making processes on their work experiences in an organization. In a similar vein, we argue that they actively infer and interpret the purpose and authentic intention of their organization’s CSR practices. This interpretation may substantially influence the relationship between CSR and OC.

For example, when an employee’s perspective taking ability is high, he or she may understand better what the purpose, true intention, and direction of the active CSR activities than an employee with low level of it. Then, he or she is likely to sincerely accept the value and purpose of CSR, perceiving that the organization not only conducts moral actions but also provides direct and indirect benefits with him or her via the CSR activities. The positive perception toward CSR may strengthen the positive influence of CSR on the employee’s attachment to the organization. In contrast, when an employee’s perspective taking ability is low, he or she cannot fully understand and accept the meaning and value of the CSR practices. Then, the positive influence of the moral activities on employees may be weakened. Therefore, this research proposes that employees’ perspective taking ability functions as an important contextual variable, which positively moderates CSR-OC link.

Taken together, we believe that this research may contribute to CSR literature in several ways. First, we investigate the influence of CSR on individual-level outcomes (i.e., employees’ perception, attitudes, and behaviors) by taking a micro-perspective. Second, we focus on an employee’s behavior (i.e., OCB) as the individual outcome. Lastly, we examine the elaborate underlying mechanism with a moderated mediation model.

## 2. Theory and Hypotheses

### 2.1. CSR and OC

Extant studies have demonstrated that CSR practices affect the level of employees’ OC [15,18,19,20]. OC is defined as a degree of organizational member’s psychological attachment to as well as will to contribute to the success of his or her organization [35,36,37]. Because CSR practices that an organization enacts provide various benefits such as training or development programs for employees, they are likely to improve the degree to which employees feel attached to their organizations in the form of OC [16]. Thus, existing studies which explain the relationship between CSR and OC tend to be grounded on social exchange theory.

The social exchange theory is based on the rule of reciprocity. That is, when someone or some group provides something valuable from the perspective of beneficiary, the beneficiary would perceive a kind of duty which he or she have to repay it in a similar way or degree [38]. For example, when an organization provides various benefits through CSR activities for its various stakeholders including community, customer, employees, and environment, the stakeholders may feel a sense of obligation to repay. Considering that an employee plays a role of both a consumer and a member of the community simultaneously, the employees are likely to perceive that he or she gets various benefits both directly and indirectly through the moral practices. Then, the employees may feel that they should repay it for the sake of their organization. Among various ways to repay it, the employees are likely to provide positive attitudes and behaviors such as OC in order to maintain balance in terms of the benefits between them and their organization. As a result, employees would increase the level of their OC. Therefore, we suggest this hypothesis

**Hypothesis** **1.** CSR is positively associated with OC.

### 2.2. OC and OCB

In this research, based on previous studies, we define OCB as an “any discretionary individual extra-role behavior advantageous to the organization” [39] (p. 284) and [40] (p. 3). OCB has been known to “shape the organizational, social, and psychological contexts that serve as the catalyst for task activities and processes” [41] (p. 100). Many previous studies have reported that OCB is closely associated with both individual-level outcomes (e.g., employee performance, turnover intentions, and absenteeism) and organizational-level outcomes (e.g., productivity and customer satisfaction) [22,23,24].

Many existing works have reported that OC is an important predictor of OCB [28,29,30,31,32]. OC is likely to increase employee’s voluntary behaviors which are not official obligations pertinent to his or her tasks, despite the fact the actions would not be rewarded by the organization [32,42]. For example, employees who strongly attached to their organization are likely to voluntarily help their organization to pursue its collective goals beyond their private interests, since they feel as if the objectives of the organization are their own [39,43]. From the perspective of the employees who have a high level of OC, helping colleagues in their organization by conducting OCB is very compatible with helping themselves, because the members in their organization play a meaningful role to define their selves [44,45]. They tend to express their commitment or loyalty to organization by providing various actions to benefit their organization in the form of extra-role behaviors (i.e., OCB).

The relationship between OC and OCB has been validated and bolstered by many previous meta-analyses [29,32,46]. The meta-analyses on the OCB–OC link have reported significant positive association between the variables. For instance, LePine and his colleagues [29] reported an effect of 0.20 for the OC–OCB link. Also, Ng and Feldman [31] demonstrated a mean correlation of 0.23 for the relationship between the OC and OCB. The results indicate that OC is closely related to OCB. Therefore, we posit this hypothesis.

**Hypothesis** **2.** An employee’s OC is positively associated with his or her OCB.

### 2.3. Mediation Effect of OC on the CSR-OCB Link

As mentioned, we posit that OC mediates the association between CSR and OCB. CSR may increase OCB via boosting the level of employees’ OC. To integrate each hypothesis based on theoretical grounds, we rely on a context–attitude-behavior framework [25,26]. The framework bolsters our overall mediation structure. This framework suggests that organizational contexts such as organizational systems or practices play an important role in influencing employees’ attitudes and their behaviors. Grounded on it, we argue that CSR may play a critical role as an organizational context which creates employee’s behavior (i.e., OCB) via affecting his or her attitude (i.e., OC). Previous studies bolster our arguments by demonstrating the positive relationship between CSR and OC [15,18,19,20], as well as OC and OCB [29,30,31,32,42]. Therefore, we hypothesize as follows.

**Hypothesis** **3.** An employee’s OC mediates the association between CSR and OCB.

### 2.4. The Moderating Effect of Employee’s Perspective Taking between CSR and OC

As described above, several studies have theoretically and empirically supported the link between CSR and OC [15,18,19,20]. Although scholars have proposed and demonstrated that CSR enhances an employee’s OC, we believe that this assumption is not always adequate to describe real pictures of an organization. Given that employees tend to actively pursue sense-making on their work experiences and interactions in an organization [47], it is hard to expect that they passively conform to the CSR practices of the organization. Instead of the simple obedience, based on their perceptions and attitudes which are originated in the sense-making processes, the employees actively interpret the intention of organizational activities (i.e., CSR). This interpretation may significantly influence the impact of the CSR on OC.

Among various potential factors to relate to the sense-making process of employees, we focus on a member’s ability of perspective taking because taking the perspective of the organization is critical for understanding purpose, authentic intentions, and value of various actions and practices of the organization. Perspective taking is defined as a cognitive procedure in which a person tries to understand others’ thoughts, intentions, preferences, and values from the viewpoint of those [33]. Through the perspective taking, the person would merge his or her mental representations with others [48]. Although the perspective taking ability can be formed by various organizational factors [49], it is widely known that people tend to be stable in the extent to which they take perspectives of others [33,34]. In other words, some individuals are more able to take the perspective of others, and are thus better at deeply understanding the target’s cognitive, emotional, and value systems. 

Thus, we expect that perspective taking is likely to help employees to understand the organization’s purpose, values, and true intention in conducting CSR practices. When an employee’s perspective taking ability is high, he or she has an enough ability to take other’s perspective. Thus, he or she is more able to understand the true intention and direction of the CSR activities. Then, he or she is likely to sincerely accept the value and purpose of CSR, perceiving that the organization not only conducts valuable things but also provides direct and indirect benefits with him or her through the CSR practices. In that case, employees are more likely to positively respond to the CSR practices in the form of enhanced perceptions, attitudes, and behaviors (i.e., OC). In other words, the perspective taking ability would boost the positive influence of CSR on OC.

**Figure 1:**
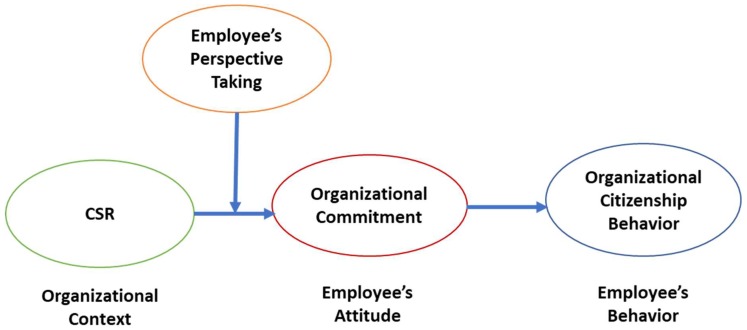
Conceptual framework of the research model.

On the contrary, when an employee’s perspective taking ability is low, he or she could not understand the true intention and values of the CSR activities from the perspective of the organization. In this situation, although an organization actively and authentically conducts CSR activities, the positive influence of CSR on employee’s OC may be weakened since the employee cannot fully translate the moral practices into organizational outcomes. When he or she can not acknowledge or even distorts the true heart of the CSR activities due to the low level of perspective taking ability, positive perceptions or attitudes toward CSR would be decreased. In that situation, the relationship between CSR and OC may not stay positive enough. Thus, we can expect that whether an employee committed to their organization through the CSR practices or not seems to depend on the extent to which an employee try to take other’s perspective at work. This is the reason why it is critical to investigate the contingent role of perspective taking in explaining the relationship between CSR and OC. In this research, we propose that perspective taking of employees may moderate the CSR-OC link (see Figure 1).

**Hypothesis** **4.** An employee’s ability of perspective taking positively moderates the relationship between CSR and an employee’s OC.

## 3. Research Methodology

### 3.1. Participants and Procedure

With an online survey system, we collected the survey data from currently working Korean employees over three different time points. The survey was conducted by one of the largest online research firms, which has the largest research panelists in South Korea (i.e., approximately 1,306,000 panelists). The research firm randomly selected the participants of our survey, thus reducing the possibility of biased sampling. By virtue of the firm’s online system, we could track down who took our survey, meaning that participants from Time 1 survey through Time 3 survey are same.

At Time 1, a total of 512 participants responded to our survey. At Time 2, 378 organizational members participated in the second survey after the first one. Also, at Time 3, 335 members responded to the third survey. The time interval between each time point was four weeks. Then, we eliminated missing data, eventually gathering data from 301 employees.

The descriptive features of the sample are as follows (see Table 1).

### 3.2. Measures

We measure our study variables with a five-point Likert scale (1 = strongly disagree, 5 = strongly agree). Then, this research computed internal consistency of the variables by utilizing Cronbach alpha values.

#### 3.2.1. CSR (Collected at Time Point 1 from Employees)

We measure CSR of each organization by utilizing 12 items of Turker’s CSR scale [50] (Cronbach alpha = 0.90). The scale is developed being grounded on the stakeholder perspective. Thus, this scale includes various dimensions which are categorized according to a variety of stakeholders. Because it was practically almost impossible to collect data from all the stakeholders, we selected four dimensions of the all stakeholders to measure the entire CSR variable: environment, community, employee, and customer dimension. Each of the selected four dimensions contains three items and represents the corresponding stakeholder of the social responsibility. For the environment dimension, sample item is “our company participates in activities which aim to protect and improve the quality of the natural environment”. For community dimension, sample item is “our company contributes to campaigns and projects that promote the well-being of the society”. For the employee dimension, sample item is “the management of our company is primarily concerned with the employees’ needs and wants”. For customer dimension, sample item is “our company respects consumer rights beyond the legal requirements”. By considering the structural position of CSR, we collected these items at Time 1.

To check whether the CSR construct has the selected four dimensions, a confirmatory factor analysis (CFA) was conducted. Then, we sequentially conduct chi-square difference tests by comparing the model fit of the four-factor model to three-factor, two-factor, and one-factor models, respectively. According to the sequential chi-square difference tests, the four-factor model (χ^2^ (df = 43) = 72.7288; CFI = 0.984; TLI = 0.975; RMSEA = 0.048) was better than the three-factor, two-factor, and single-factor model.

#### 3.2.2. OC (Time Point 2, Collected from Employees)

OC was measured by four items of Meyer and Allen’s scale [35] at Time 2 (Cronbach alpha = 0.88). Sample items were (a) “I really feel as if my organization’s problems are my own”; (b) “I feel a strong sense of belonging to my organization”; (c) “I feel emotionally attached to my organization”.

#### 3.2.3. OCB (Time Point 3, Collected from an Immediate Leader of Employees)

At Time 3, we utilized the evaluations of an immediate leader of each employee to measure the level of employee’s OCB. The measure consists of five items from Spector and his colleagues’ OCB scale [51]. Sample items are “This employee helped a co-worker who had too much to do”; “This employee helped new employees get oriented to the job”; and “This employee lent a compassionate ear when someone had a work problem”. We expect that collecting data from multi-source will decrease the potential problems of common method bias (Cronbach alpha = 0.90).

#### 3.2.4. Perspective Taking (Time Point 1, Collected from Employees)

To measure the level of employees’ perspective taking ability, we used four items of perspective taking scale which was developed by Davis and his colleagues [48]. Sample items are “I made an effort to see the world through my coworkers’ eyes”; “I sought to understand my coworkers’ viewpoints”; “I tried to take my coworkers’ perspectives”. The value of Cronbach alpha in this study was = 0.84.

#### 3.2.5. Control Variables

We control several variables for OCB to reduce the bias during the estimation processes. OCB is controlled by organization tenure, gender, position, and education level [52,53]. For the consistency of our research, we gathered the control variables at Time 2.

### 3.3. Statistical Analysis

For the baseline statistics, a correlation analysis is conducted to our data. Considering that the hypothesized model of our research contains multiple variables, we use SEM to analyze the moderated mediation model and obtain the fit indices of the model [54]. According to Anderson and Gerbing [55], we conduct a two-step approach in which the measurement model is tested first and then the structural model is tested after that. We adopt several goodness-of-fit indices and their own criteria which are suggested in the previous literature. Desirable fit indices are associated with a CFI and a TLI greater than 0.90, and a RMSEA less than or equal to 0.06 [56].

After obtaining the model fit indices, we conduct chi-square difference tests by comparing the hypothesized model with a nested alternative model [57]. Through this comparison test, we can find the model with the best goodness-of-fit indices.

Finally, we conduct a bootstrapping analysis in order to examine whether OC mediates the relationship between CSR and OCB [58]. Together with the test for mediation effect, we also examine whether PT moderates the influence of CSR on OC with the moderated mediation model using SEM [59].

## 4. Results

### 4.1. Descriptive Statistics

To explore the statistical features of the variables, we calculate the means and standard deviations of each variable incorporated in our research. Pearson correlation coefficients are also calculated for each pair of the variables. The descriptive statistics are shown in Table 2.

As for position, general manager or higher are coded as 5, deputy general manager and department manager 4, assistant manager 3, clerk 2, and others below clerk as 1. As for education, “below high school diploma” level is coded as 1, “community college” level as 2, “bachelor’s” level as 3, and “master’s degree or more” level is coded as 5.

### 4.2. Measurement Model

We perform a confirmatory factor analysis (CFAs) for all our research variables in order to examine the goodness-of-fit of the measurement model (see Table 3 and Table 4). Since three psychometric constructs (i.e., CSR, OC, and PT) are incorporated in our search, discriminant validity of the three variables is identified. In this measurement model, we considered the CSR as a variable which consists of four sub-dimensions (i.e., CSR for environment, community, customer, and employees), based on the above CFA analysis for the CSR variable. The three-factor model turns out to show a good fit to the observations (χ^2^ (df = 48) = 75.30; CFI = 0.984; TLI = 0.978; RMSEA = 0.044). Additionally, we conduct chi-square (χ^2^) difference tests by sequentially comparing the three-factor model with two-factor and single-factor models. The chi-square difference test reveals that the three-factor model shows better fit to the observed data than other (i.e., two-factor and single-factor) models. Thus, we confirm the three variables can be distinct.

### 4.3. Structural Model

#### 4.3.1. Result of Mediation Analysis

We build a structural equation model, so-called ‘moderated mediation model’, including mediating and moderating structures between CSR and OCB at the same time. In the mediating structure, the link between CSR and OCB is mediated by OC. In the moderating structure, PT moderates the effect of CSR to OC.

Prior to other analyses, we transform the variables into the mean-centered ones. Note that centered variables are useful in (i) estimating the interaction terms without any expense of correlations and (ii) reducing and testing multicollinearities among the variables. Afterwards, we calculate the interaction term by multiplying the centered perceived CSR and PT.

In addition, we test whether there is a multicollinearity bias between the independent variables (CSR and PT) by utilizing SPSS. As a result, we obtain the variance inflation factors (VIF) and tolerances to test multicollinearity [60]. The VIF for CSR and PP are 1.12 and 1.12, respectively, and the tolerance statistics are 0.89 and 0.89, respectively. Since the obtained VIF values are all sufficiently smaller than 10, combined with the tolerance statistics above 0.2, we can conclude that the two variables (CSR and PT) do not have an issue of multicollinearity.

Then, we conduct SEM analyses and chi-square difference tests between the hypothetical model and alternative nested model. The full mediation model of our analysis (Model 1) is tested and the fit indices are obtained to be acceptable: χ^2^ = 223.828 (df = 131), CFI = 0.960, TLI = 0.948, and RMSEA = 0.049. An alternative nested model of our analysis (Model 2) is a partial mediation model which contains a direct path from CSR to OCB. The fit indices of Model 2 are also good enough: χ^2^ = 213.611 (df = 130); CFI = 0.964; TLI = 0.953; RMSEA = 0.046. The chi-square difference test for Model 1 and Model 2 tells us that the partial mediation model (Model 2) rather than the full mediation model (Model 1) shows a better fit (Δχ^2^ [1] = 10.217, *p* < 0.01).

**Figure 2:**
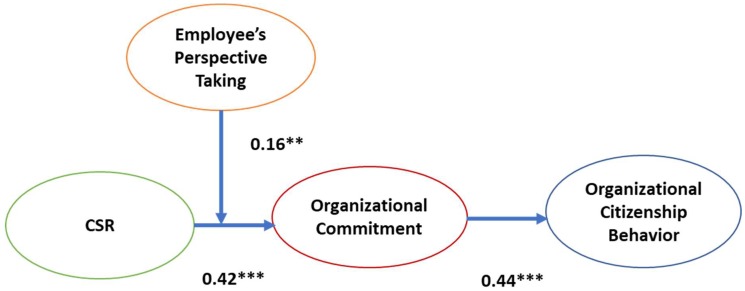
Final model (based on the findings) Notes: ** *p* < 0.01, *** *p* < 0.001.

Figure 2 presents the best-fitting, hypothesize model. The control variables including position, tenure, and education level turn out to be statistically non-significant, except for gender. Incorporating the control variables, our model shows that CSR is significantly associated with OC (β = 0.42, *p* < 0.001), supporting Hypothesis 1 and that OC is significantly associated with OCB (β = 0.44, *p* < 0.001), supporting Hypothesis 2.

#### 4.3.2. Result of Moderation Analysis

**Figure 3:**
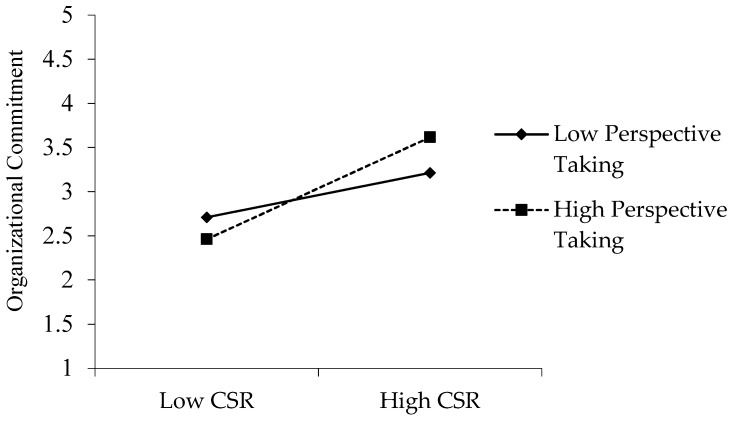
Moderating effect of PT on the relationship between CSR and OC.

The moderation effect of PT on the association between CSR and OC is tested by the moderated mediation model (see Figure 3). As already mentioned, CSR and OC are transformed into the mean-centered form and the interaction term is obtained by multiplying the two transformed variables [59]. The coefficient of the interaction term (β = 0.16, *p* < 0.01) turns out to be significant which implies that there exists the moderating effect of PT on the association between CSR and OC. In other words, when the level of PT is high, the positive effect of CSR on OC is amplified, which supports Hypothesis 4.

### 4.4. Bootstrapping

Bootstrapping procedures are conducted by using a sample of 5000 [58] in order to test Hypothesis 3 which suggests the mediating role of OC between CSR and OCB. Note that the indirect mediation effect is significant at 5% level if the 95% bias-corrected confidence interval (CI) for the mean indirect mediation effect excludes zero [58]. In our analysis, the bias-corrected CI for the mean indirect effect on the path, which is from CSR via OC to the OCB, does not include zero (95% CI = [0.11, 0.45]). Hence, this suggests that the indirect mediation effect of OC on the path from CSR to OCB is significant at level of 5%, supporting Hypothesis 3.

## 5. Discussion and Conclusions

In the present study, we try to reveal the intermediating process of the relationship between CSR and OCB. To test our hypotheses, we utilized three-wave time-lagged survey data from organizational members in various firms of South Korea. By conducting a moderated mediation model analysis with SEM technique, we examined the underlying mechanism that intermediates the CSR-OCB link as well as an important contextual factor which moderates the relationship. Our results show that not only an employee’s OC functions as a mediator between CSR and OCB, but also employee’s perspective-taking ability positively moderates the CSR-OC link. We discuss various implications of our findings from the perspective of theory and practice. Limitations and directions for future studies were also described.

### 5.1. Theoretical Implication

We expect that this paper would contribute to expanding CSR literature by providing some theoretical implication. First, we focus on the effect of CSR on organizational outcomes at the individual level (e.g., employee’s perceptions, attitudes, and behaviors toward CSR). The previous works on CSR have mainly examined organizational outcomes at the macro-level (e.g., financial performances, product quality, reputation of firm, consumer loyalty, and consumer evaluation of product/company) by delving into the effects of CSR on external stakeholders (i.e., shareholders, customers, and local communities). Thus, preceding studies have relatively underexplored the relationship between CSR and organizational outcomes at the individual level [2,13,14]. Considering that employees are the very agent who substantially translate moral endeavors such as CSR activities into organizational outcomes [2], the perceptions, attitudes, and behaviors of employees toward CSR are critical to maximizing the positive influence of CSR in an organization.

Second, although some existing works have delved into the association between CSR and organizational outcomes at the individual level, those studies have paid relatively less attention to the behaviors of employees. Given that employee’s behaviors not only reflect their perceptions and attitudes, but also tend to more directly explain the macro-level organizational outcomes such as financial performance, investigating the CSR-employee’s behavior link is critical to understand the influence of CSR in an organization. Thus, our attempt to investigate the influence of CSR on employee’s behavior (i.e., OCB) would contribute to CSR literature.

Third, as Aguinis and Glavas [2] suggested, previous studies on CSR did not relatively pay enough attention to underlying processes in CSR-organizational outcomes link. In specific, research which investigated the influence of CSR on OCB in an elaborate way is very scarce [61,62,63]. To complement those research gap, we examined intermediating process in the association. Specifically, we revealed that OC mediates the CSR-OCB link. Relied on theoretical and empirical evidence, this research would contribute to CSR research by providing the elaborate mechanism of the relationship.

Lastly, by delving into moderating effect of employee’s perspective taking ability in the CSR-OC link, we revealed the contextual variable which moderate the influence of CSR on employees’ attitudes (i.e., OC). We believe this approach is reasonable since employees’ sense-making processes on their work experiences are critical for them in interpreting experiences about CSR in an organization [47]. Among a variety of factors, we focus on employees’ perspective taking ability which functions as an important contextual variable that positively moderates the CSR-OC link.

### 5.2. Practical Implications

Our findings also have practical implications for practitioners in an organization. First, this research may provide leader with insights about underlying process between CSR and OCB. The results showed that CSR practices increase the quality of employees’ OCB through boosting employees’ OC. Therefore, a top management team or managers who attempt to enhance employees’ work-related behaviors (i.e., extra-role behaviors) through conducting CSR activities should closely watch the changes in individual employees’ attitudes such as OC. If the employees did not present signs of increased OC, it means that the organization’s CSR practices do not effectively work enough to boost OCB. Considering our theoretical model which explains the underlying process between CSR and OCB, top management teams in an organization had better observe and manage employees’ attitudes that intermediate the link.

Second, our results demonstrated that employees’ perspective taking ability positively moderates the association between CSR and OC. We believe that the results would emphasize the significance of employees’ characteristics in increasing the benefit of CSR activities. As our findings revealed, CSR activities itself are not enough to fully facilitate employees’ OC. Its positive influence would be maximized when employees are more able to understand the organization’ values and true intention which are reflected in the form of CSR. Thus, we suggest that top management teams should attempt to foster perspective taking ability of employees.

### 5.3. Limitations and Suggestions for Future Studies

Although we believe that this research has valuable implications from the theoretical and empirical point of views, it has some limitations which need to be addressed. First, considering that employees’ perceptions or attitudes may be influenced by their cultural characteristics, we should consider cultural differences in terms of employees’ attitudes toward the CSR activities, despite its universal values regardless of Western and Eastern culture [64,65]. Therefore, we should be cautious to apply our findings to another context. In addition, since South Korea not only experienced very rapid economic growth, but has also been embedded in the collectivistic culture [66], we believe that future research had better consider these cultural issues.

Second, we acknowledge that this study is influenced by common method bias since employees responded to CSR, OC, and perspective-taking, scales. Although not only the OCB scale was measured by an immediate leader of the employees, but also the result of CFA bolsters the distinctiveness among the variables, future studies should adequately deal with this issue.

Although the present paper includes various limitations, we believe that it may deepen the CSR literature by investigating an underlying process as well as a contingent variable through which CSR influences OCB. Our findings show that an employee’s OC plays an intermediating role which connects CSR with OCB. In addition, we also found the significance of an employee’s perspective ability to maximize the positive effects of CSR on employee’s attitudes.

## Figures and Tables

**Table 1:** Descriptive Characteristics of our sample.

Characteristic	Percent
**Gender**	
Male	48.2%
Female	51.8%
**Age**	
20s	21.6%
30s	24.9%
40s	25.9%
50s	27.6%
**Occupation**	
Office workers	62.1%
Administrative positions	19.9%
Sales & marketing	6.0%
Manufacturing worker	5.3%
Education	2.0%
**Position**	
Staff	29.2%
Assistant manager	25.2%
Manager or deputy general manager	30.3%
Department/general manager and above director	15.3%
**Tenure (in month)**	
Below 50	52.2%
50 to 100	18.6%
100 to 150	13.9%
150 to 200	5.0%
200 to 250	4.7%
Above 250	5.6%
**Firm size**	
Above 500 members	19.6%
300–499 members	6.0%
100–299 members	15.6%
50–99 members	13.3%
Below 50 members	45.5%
**Industry Type**	
Manufacturing	24.6%
Services	13.6%
Construction	12.6%
Information service and telecommunications	10.3%
Education	10.0%
Health and welfare	8.3%
Public service and administration	7.3%
Financial/insurance	3.7%

**Table 2:** Means, standard deviations, and inter-correlations of measures.

	Mean	SD	1	2	3	4	7	8	9
1. Gender_T2	1.52	0.50	-						
2. Position_T2	2.55	1.38	−0.36 **	-					
3. Tenure (Months)_T2	79.66	82.04	−0.11 *	0.32 **	-				
4. Education_T2	2.58	0.83	−0.07	0.17 **	0.00	-			
7. CSR_T1	3.20	0.61	−0.10	0.13 *	0.20 **	−0.03	-		
8. PT_T1	3.60	0.57	0.02	0.09	0.05	0.07	0.33 **	-	
9. OC_T2	3.00	0.82	−0.05	0.24 **	0.18 **	0.03	0.41 **	0.24 **	-
10. OCB_T3	3.19	0.69	0.10	0.11	0.11	0.00	0.34 **	0.27 **	0.47 **

Note: * *p* < 0.05. ** *p* < 0.01. As for gender, males are coded as 1 and females as 2.

**Table 3:** Chi-square difference tests among alternative measurement models.

Model	χ^2^	*df*	CFI	TLI	RMSEA	Δ*df*	Δχ^2^	Preference
1 Factor Model	637.970	51	0.657	0.557	0.196			
2 Factor Model	553.559	50	0.706	0.612	0.183	1	84.411	2 Factor Model
3 Factor Model	75.30	48	0.984	0.978	0.031	2	478.259	3 Factor Model

**Table 4:** Result of CFA for measurement model including factor loading per item.

Measurement Model	Unstandardized Coefficient	Standardized Coefficient	*t*-Value
CSR → CSR for Environment	1	0.629	
CSR → CSR for Community	1.206	0.679	11.260 ***
CSR → CSR for Customer	1.104	0.708	6.787 ***
CSR → CSR for Employee	1.440	0.802	7.206 ***
PT → PT 1	1	0.750	
PT → PT 2	1.009	0.708	11.510 ***
PT → PT 3	1.100	0.766	12.418 ***
PT → PT 4	1.030	0.818	13.068 ***
OC → OC 1	1	0.781	
OC → OC 2	0.970	0.676	14.402 ***
OC → OC 3	1.276	0.851	15.252 ***
OC → OC 4	1.227	0.878	15.549 ***

PT means perspective taking, OC means organizational commitment. *** *p* < 0.001.
